# Fracture of the Tibial Baseplate in Bicompartmental Knee Arthroplasty

**DOI:** 10.1155/2015/693025

**Published:** 2015-12-30

**Authors:** Bart Stuyts, Melanie Vandenberghe, Hans Van der Bracht, Yves Fortems, Elke Van den Eeden, Luc Cuypers

**Affiliations:** ^1^Sint-Augustinus Hospital, 2610 Wilrijk, Belgium; ^2^Sint-Jozef Hospital, 2390 Malle, Belgium; ^3^Antwerp University Hospital, Wilrijkstraat 10, 2650 Edegem, Belgium

## Abstract

*Introduction*. Bicompartmental knee arthroplasty (BKA) addresses combined medial and patellofemoral compartment osteoarthritis, which is relatively common, and has been proposed as a bridge between unicompartmental and total knee arthroplasty (TKA).* Case Presentation*. We present the case report of a young active man treated with BKA after unsuccessful conservative therapy. Four years later, loosening with fracture of the tibial baseplate was identified and the patient was revised to TKA.* Discussion*. Although our case is only the second fractured tibial baseplate to be reported, we believe that the modular titanium design, with two fixation pegs, is too thin to withstand daily cyclic loading powers. Light daily routine use, rather than high-impact sports, is therefore advised. Failures may also be related to the implant being an early generation and known to be technically complex, with too few implant sizes. We currently use TKA for the treatment of medial and patellofemoral compartment osteoarthritis.

## 1. Introduction

Total knee arthroplasty (TKA) is the gold standard treatment for patients with end-stage knee osteoarthritis (OA). However, it is considered too drastic solution in younger patients with isolated medial and patellofemoral compartment OA [1]. For isolated medial or lateral compartment OA, an alternative is unicondylar knee arthroplasty (UKA), which retains the cruciate ligaments intact and allows for minimal surgical exposure.

Bicompartmental knee arthroplasty (BKA) was developed to bridge the gap between UKA and TKA. BKA addresses combined medial and patellofemoral compartment OA, which are commonly affected by OA [2, 3]. The prosthesis consists of a femoral shield with a medial condylar resurfacing component and a fixed-bearing unicondylar tibial knee prosthesis. BKA keeps the cruciate ligaments and lateral compartment intact [1] and mimics natural knee translation and rotation during weight bearing [1]. BKA has a number of advantages over TKA, including more physiological tibiofemoral kinematics and enhanced stability [4] and proprioception [5, 6]. The BKA procedure minimizes blood loss and shortens postoperative hospital stays, which facilitates faster patient recovery [7, 8].

We here present the case of an active young man who was treated with a BKA and developed a rare complication.

## 2. Case Presentation

A 52-year-old male patient with no significant medical history presented to our outpatient clinic complaining of soreness in the left knee during jogging. He jogged for several hours per week, with progressively worsening pain. The knee was stable and had full range of motion, with little hydrops, no meniscal irritation, and a mild varus angulation. X-rays were taken and medial gonarthrosis was diagnosed.

**Figure 1:**
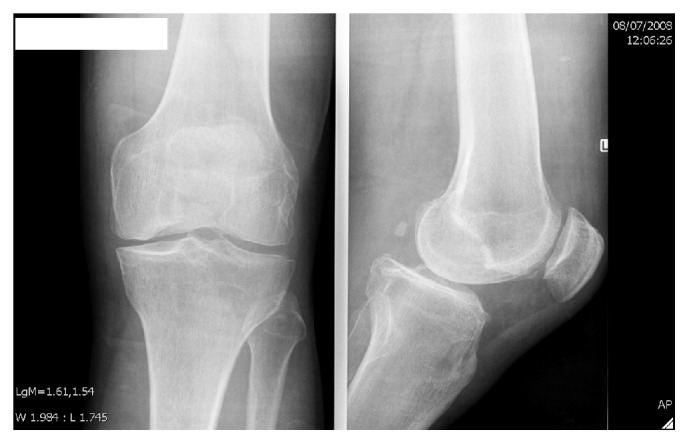
Anteroposterior and profile view X-rays of the knee.

Taking into account his young age and mild symptoms, conservative treatment was initiated. The patient was advised to cut back on his training and take a glucosamine-chondroitin supplement for 3 months. When the complaint did not get better, an intra-articular cortisone injection was administered. Further X-rays revealed osteochondritis dissecans of the medial condyle (Figure 1). The medial femoral condyle was found to have grade 4 cartilage defects covering 70% of the surface on arthroscopic evaluation. The patellofemoral compartment had grade 2-3 cartilage defects. The cruciate ligaments, menisci, and the lateral compartment were normal and intact.

As a result of the scale of his complaints and the need to stop all sports, the patient requested a more permanent solution. To address his extensive medial compartment damage and advanced symptomatic patellofemoral chondropathy, a Deuce Journey (Smith & Nephew Inc., Memphis, TN, USA) bicompartmental arthroplasty of the left knee was proposed. A HTO was considered, but because of the grade IV degeneration of the medial compartment, it was not our treatment of choice as severe degenerative involvement of the medial compartment still remains a contraindication for HTO [9]. Following a classic midline parapatellar incision, the patellar osteophytes were denervated and resected. The medial meniscus was then resected and femoral and tibial cuts were made. A size-8 femoral component, a size-6 tibial component, an 8 mm meniscal insert and a size-29 cemented patella were implanted. Stable fixation and excellent patellar tracking were achieved.

**Figure 2:**
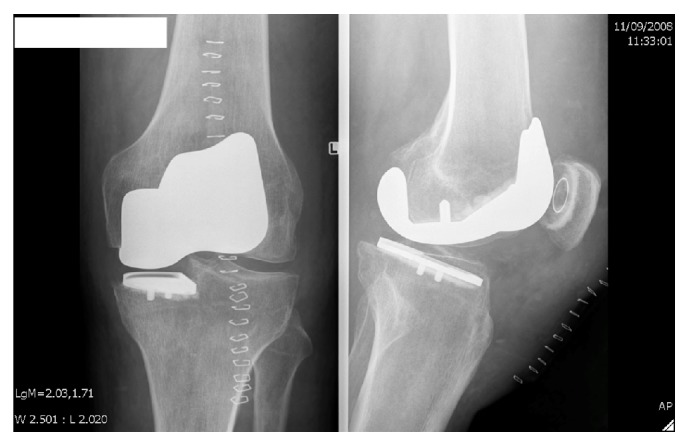
Anteroposterior and profile view X-rays of the knee following surgery.

Postoperative care followed a standardized protocol and was uncomplicated. Six-week follow-up with X-rays (Figure 2) was reassuring and the patient resumed exercise 3 months after the procedure. Four-month and 1-year postoperative outpatient visits revealed no objective or subjective complaints other than a sore knee after extensive running. The X-rays remained reassuring.

**Figure 3:**
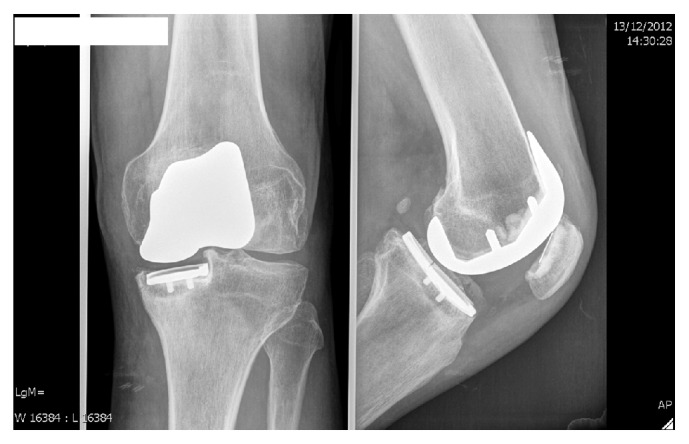
Anteroposterior and profile view X-rays of the knee 4 years after surgery. Arrow indicates fracture of the baseplate.

**Figure 4:**
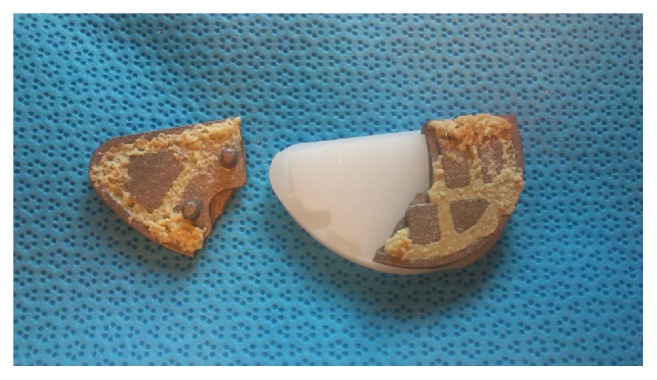
The fractured tibial baseplate.

**Figure 5:**
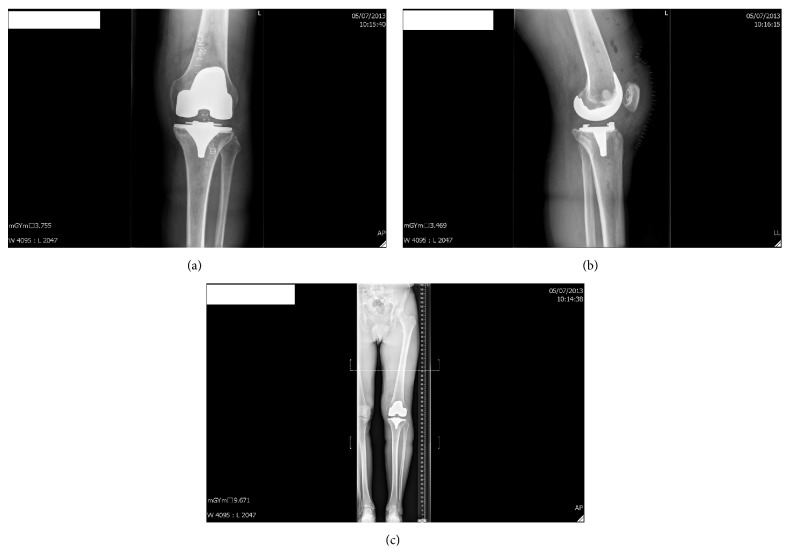
Anteroposterior (a), profile (b), and full leg view (c) X-rays of the knee after revision surgery.

During routine consultation 4 years following surgery, the patient complained of increasing pain on the medial side of the knee while running. Normal daily activities gave no pain. Clinical investigation revealed a normal range of motion in a stable knee, no hydrops, and a little pressure soreness over the medial tibial plateau. On X-ray examination, subsidence and fracture line of the tibial baseplate were visible (Figure 3), while a bone scan revealed hypercaptation of the tibial baseplate. C-reactive protein (CRP) levels remained below baseline and aspiration was negative. A diagnosis of loosening with fracture of the tibial baseplate (Figure 4) was made and the patient was revised to TKA almost 5 years following the index procedure (Figure 5).

## 3. Discussion

Bicompartmental OA of the knee is not rare, and young and active patients desire a rapid and seamless return to their former lifestyle, with full function and no pain. UKA and BKA can prevent or postpone TKA and preserve the bone stock and restore more normal kinematics [1, 10–14]. The functional status of the patient is also improved by leaving the anterior cruciate ligament intact [15–18], while maintaining the proprioceptive influence of the cruciate ligaments gives a better functional result for the patient with advanced OA, with improved overall patient satisfaction and stair climbing [1, 19, 20]. The shortcomings of BKA are those of UKA: it is unable to correct severe deviations of the mechanical axis and requires intact ligamentous structures. Patellofemoral OA is not a contraindication for UKA, but it should be no more than grade II according to Iwano et al. and asymptomatic. Anterior knee pain with secondary patellofemoral joint degeneration should be excluded during patient selection [21, 22].

There are few alternatives to the BKA other than performing UKA at an earlier stage and converting to a TKA when necessary. Although this appears acceptable, conversion of UKA to TKA is associated with a poorer clinical outcome than primary TKA [23].

An alternative to a monoblock BKA is a BKA with two independent implants (UKA/patellofemoral joint replacement), because the anatomy and orientation of the condyles and the trochlea are not standard but related to morphotype, gender, and race. Consequently, the extreme variability in their dimension, and in the distance and angle between the axis of the condyles and of the trochlea, often necessitates a “custom-made” replacement. This may be achieved through the use of two independent small implants [22]. These types of procedures are technically demanding with a survival rate of only 54% at 17 years of follow-up. The high revision rate, compared with total knee arthroplasty, may be related to several factors such as implant design, patient selection, crude or absent instrumentation, or component malalignment, which can all contribute to the relatively high failure rate [24]. With more modern instrumentation, including the aid of a robot, the results seem to be more promising with 83% good to excellent results at a mean follow-up of 27 months [25].

While another option is primary TKA, the OA and associated complaints are not always so severe or advanced to warrant this more invasive procedure. There are also very few cases of progressive lateral OA, thus confirming the indication for bicompartmental arthroplasty.

Outcome studies of the success and revision rates of BKA are rare. Some studies indicate that although both BKA and TKA result in reduced pain and improved physical function in the early postoperative period, the benefit is greater with BKA, with a more rapid and marked reduction in stiffness [18]. However, this difference does not persist beyond 1 year postoperatively [26]. Other studies suggest a high rate of early complications with BKA, such as persistent pain, which could require revision arthroplasty [26], and early failure [27]. Palumbo et al. investigated the Deuce Journey BKA in 32 patients, reporting inconsistent pain relief and functional results, with a survival rate of 86% and one failed tibial baseplate [28]. The patient was converted to TKA 15 months after the index procedure.

Although ours is only the second fractured tibial baseplate to be reported in the literature, we found the modular titanium design, with two fixation pegs, to be somewhat thin to withstand daily cyclical loading powers. We believe that fracture of the tibial baseplate following BKA must therefore be considered as a possible complication and, following implantation, light daily routine use is probably advisable rather than the pressures that high-impact sports can produce. Hopper and Leach reported that patients had a significantly greater return to sport rate after UKA than patients who had undergone TKA [29]. A large proportion of patients in the TKA group did not return to sport, which their surgeon would have expected them to including golf and bowls. Patients in the UKA group were more involved in sport activities than patients in the TKA group. Moreover, patients undergoing UKA also returned to sport more quickly than patients undergoing TKA [29]. The majority of patients returned to sports and recreational activity after unicompartmental knee arthroplasty. The most common activities after surgery were hiking, cycling, and swimming. Several high-impact activities as well as the winter disciplines of downhill and cross-country skiing had a significant decrease in participating patients. The majority of the patients (90.3%) stated that surgery had maintained or improved their ability to participate in sports or recreational activities [30].

Failures may also be related to the implant being an early generation that is known to be technically complex, with an insufficient range of implant sizes. Taken together, these factors increase the risk of malalignment and instability [27].

Fracture of the metallic components is a rare but potential cause of failure of unicompartmental knee arthroplasty. In the experience of Manzotti et al., the incidence of this complication was 4.9% (6/121) of all UKA failures [31]. Only 2/121 (1.6%) were fractures of the tibial base plate. Patients with a BMI greater than 30 and a progressive deterioration in limb alignment were at greater risk [31].

With exact anatomical positioning, the Journey Deuce prosthesis achieves good functional outcomes, which could be obtained more easily with a wider range of implant sizes. This would make the BKA a viable option for knee arthroplasty, as partial knee replacement with retained anatomical function and reduced bone loss remains an important concept. For the moment, we have chosen to perform TKA for the treatment of medial and patellofemoral compartment OA.
